# Impact of the COVID-19 pandemic on hospital episodes for falls and fractures associated with new-onset disability and frailty in England: a national cohort study

**DOI:** 10.1093/ageing/afae071

**Published:** 2024-04-06

**Authors:** Seth Thomas, Kathryn Littleboy, Josephine Foubert, Vahe Nafilyan, Neil Bannister, Ash Routen, Richard Morriss, Kamlesh Khunti, Natalie Armstrong, Laura J Gray, Adam L Gordon

**Affiliations:** Data and Analysis for Social Care and Health, Health Analysis and Pandemic Insights, Office for National Statistics, Newport, UK; Data and Analysis for Social Care and Health, Health Analysis and Pandemic Insights, Office for National Statistics, Newport, UK; Data and Analysis for Social Care and Health, Health Analysis and Pandemic Insights, Office for National Statistics, Newport, UK; Data and Analysis for Social Care and Health, Health Analysis and Pandemic Insights, Office for National Statistics, Newport, UK; Data and Analysis for Social Care and Health, Health Analysis and Pandemic Insights, Office for National Statistics, Newport, UK; NIHR Applied Research Collaboration East Midlands, Leicester, UK; Diabetes Research Centre, University of Leicester, Leicester, UK; NIHR Applied Research Collaboration East Midlands, Leicester, UK; Institute of Mental Health, University of Nottingham, Nottingham, UK; NIHR Applied Research Collaboration East Midlands, Leicester, UK; Diabetes Research Centre, University of Leicester, Leicester, UK; NIHR Applied Research Collaboration East Midlands, Leicester, UK; Department of Population Health Sciences, University of Leicester, Leicester, UK; NIHR Applied Research Collaboration East Midlands, Leicester, UK; Department of Population Health Sciences, University of Leicester, Leicester, UK; NIHR Applied Research Collaboration East Midlands, Leicester, UK; Academic Unit of Injury, Recovery and Inflammation Sciences (IRIS), School of Medicine, University of Nottingham, Nottingham, UK; Department of Medicine for the Elderly, University Hospitals of Derby and Burton NHS Foundation Trust, Derby, UK

**Keywords:** falls, fractures, COVID-19, frailty, older people

## Abstract

**Background:**

Older people with frailty are at risk of harm from immobility or isolation, yet data about how COVID-19 lockdowns affected them are limited. Falls and fractures are easily measurable adverse outcomes correlated with frailty. We investigated whether English hospital admission rates for falls and fractures varied from the expected trajectory during the COVID-19 pandemic, and how these varied by frailty status.

**Methods:**

NHS England Hospital Episode Statistics Admitted Patient Care data were analysed for observed versus predicted outcome rates for 24 January 2020 to 31 December 2021. An auto-regressive integrated moving average time-series model was trained using falls and fracture incidence data from 2013 to 2018 and validated using data from 2019. Models included national and age-, sex- and region-stratified forecasts. Outcome measures were hospital admissions for falls, fractures, and falls and fractures combined. Frailty was defined using the Hospital Frailty Risk Score.

**Results:**

144,148,915 pre-pandemic hospital admissions were compared with 42,267,318 admissions after pandemic onset. For the whole population, falls and fracture rates were below predicted for the first period of national lockdown, followed by a rapid return to rates close to predicted. Thereafter, rates followed expected trends. For people living with frailty, however, falls and fractures increased above expected rates during periods of national lockdown and remained elevated throughout the study period. Effects of frailty were independent of age.

**Conclusions:**

People living with frailty experienced increased fall and fracture rates above expected during and following periods of national lockdown. These remained persistently elevated throughout the study period.

## Key points

- Falls and fractures are important proxy markers of disability and frailty.
- For the whole population, falls and fracture rates were below predicted for first national lockdown, then returned to predicted.
- For people with frailty, the risk of falls and fractures rose after lockdown periods and remained elevated, unlike other population groups.
- People with frailty deserve specific consideration in pandemic planning if lasting harms are to be mitigated against.

## Introduction

Older people [1], ethnic minority groups [2], people living with disability [3] and those experiencing socioeconomic deprivation [4] were disproportionately burdened by the COVID-19 pandemic through direct impacts including increased risk of hospitalisation and mortality from SARS-CoV-2 infection, and indirect impacts including social isolation [5].

In England, 43% of hospital admissions and 68% of those dying with COVID-19 during the pandemic were aged ≥75 years [6, 7]. Older people were more likely to experience social isolation because of increased prevalence of functional dependency, frailty and multiple long-term conditions with age [8]. Older people were also less likely to be able to conduct activities of daily living without assistance and more likely to have clinical extremely vulnerable status—the basis of government recommendations for extended or enhanced social isolation [9].

Social isolation is associated with multiple harms, including increased prevalence of mental health diagnoses and loneliness [10]. Self-reported wellbeing data collected during the pandemic showed older people experienced physical deconditioning associated with social isolation [10]. Deconditioning describes deterioration in physical performance and is an important precursor to new-onset disability and frailty [11]. There is real concern that the pandemic may have increased incidence of disability and frailty, as with increases in cardiovascular [12] and cancer-related [13] morbidity and mortality, due to altered health and healthcare-seeking behaviour.

Frailty and transition into disability are not routinely recorded in UK population health or health utilisation data. Falls and fractures are both associated with disability and frailty, and can be used as proxy measures of frailty prevalence at a population level [14]. Almost all falls are multifactorial and are more frequent in people living with frailty and disability due to failure of one or more homeostatic mechanisms regulating gait and posture [15]. Fractures are a direct consequence of falls, including some 80,000 hip fractures and 200,000 non-hip fractures in the UK per annum [16]. Hip fractures in particular are associated with frailty. The average hip fracture patient is 82.5 years old [17], with a mean (SD) admission frailty index of 0.34 (0.16) [18], indicating moderate frailty.

We aimed to establish whether admissions to hospitals in England with falls and fractures varied significantly from their expected trajectory during the COVID-19 pandemic, by comparison with pre-pandemic rates established over the preceding 10 years, and how these outcomes varied by frailty status. We also considered whether geographical differences in pandemic isolation were associated with falls and fractures as markers of new-onset disability and frailty.

## Methods

### Data

We extracted data from NHS England’s Hospital Episode Statistics Admitted Patient Care (HES APC) database using International Classification of Diseases and Related Health Problems 2010 (ICD-10) codes for pre-pandemic (1 January 2013 to 23 January 2020) and during pandemic (24 January 2020 to 31 December 2021) years. A total of 144,148,915 hospital records were available for extraction for pre-pandemic and 42,267,318 records after pandemic onset.

Three clusters of ICD-10 diagnosis codes were of interest: Fractures, Falls and Frailty. Fracture clusters comprised a subset of codes from Chapter 19 of the ICD-10 (2019) focused on fractures commonly experienced by older people (Supplementary materials, Table S1). Fall clusters selected for ICD-10 codes W00–W19. Frailty clusters selected for codes included in the Hospital Frailty Risk Score (HFRS) [19]. Hospital episodes were selected based on presence of these codes in primary or secondary diagnosis fields of APC records. A hospital episode was classified as a frail fall and fracture, if an individual had HES record criteria for frailty based upon a Hospital Frailty Risk Score ≥5 [19] and a concurrent fall and fracture recorded on the same episode record. Episode data were binned to monthly frequency for time-series modelling.

### Statistical modelling

Auto-regressive integrated moving average is a commonly used statistical technique for time-series modelling. Such models have demonstrated strong predictive capability in the field of public health [20, 21] with proficient ability to create short-term forecasts.

Time-series modelling accuracy is typically assessed stepwise, validating the predicted value against known observations. The onset of the pandemic precluded forecasted values from such validation techniques. Model accuracy was therefore assessed prior to pandemic onset using a validation period of 2018–2019. Accuracy was determined across a 5-year training period from 2013, in a monthly stepwise manner using root-mean-squared error and mean absolute percentage error metrics. Models that minimised these error metrics were deemed most suitable for generating pandemic year forecasts.

Models were optimised for national, age-stratified, sex-stratified and region-stratified forecasts for each respective episode group. Age groups were split into four categories and enumerated at hospital episode age: children (≤18 years), working-age adults (19–64 years inclusive), pension-age adults (65–79 years inclusive) and oldest old (≥80 years). For region stratification, episodes were filtered to International Territorial Level regions (e.g. North West). Some 69,944 episodes records were lacking regional information and were excluded from regional stratification analysis. All analyses were conducted in Cloudera Data Science Workbench using R (version 3.5.1).

Aggregated models were assessed for accuracy using low mean absolute percentage error (MAPE) metrics. MAPE measures the average magnitude of error produced by a model, or how far off predictions are on average, by summing absolute percent errors together and dividing the sum by the number of errors.

## Results

Episode rates were calculated using Office of National Statistics (ONS) annual population estimates to express the number of hospital episodes of interest per 100,000 people (Table 1). In this table, and in subsequent analysis, falls and fractures are for the population as a whole, including those found to have frailty. Frail falls and fractures is for a subset of the population.

**Table 1:** Episode incidence rates per 100,000 population for fractures, falls, and frail falls and fractures for all of England

Year	Fractures	Falls	Frail falls and fractures
2013	260.07	297.22	47.44
2014	261.99	312.55	47.29
2015	260.23	318.05	46.44
2016	264.79	325.99	46.7
2017	275.94	337.68	47.59
2018	289.98	354.77	49.31
2019	295.22	369.47	50.81
2020	277.74	380.73	54.34
2021	292.31	388.27	55.65

Incidence rates decreased for fracture episodes in the first year of the pandemic in contrast to falls and frail fall and fractures, where the largest increase in incidence rates was observed. An increase in incidence rates was evident for all outcomes in 2021, though this remained lower than pre-pandemic rates for fractures. The absolute difference between expected and observed admissions per indication were as follows: fractures 27,394, falls 13,921, and frail falls and fractures 3,461—with episodes in each category being lower than expected overall.

**Figure 1:**
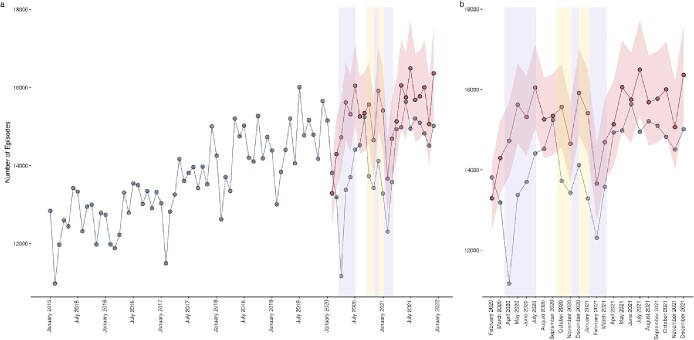
Actual versus forecasted fracture episodes for all of England. Blue data series indicate observed episodes, red data series indicate estimated episodes and their 95% confidence intervals, lilac shaded areas indicate periods of national lockdown restrictions and yellow shaded areas indicate periods of local tiered restrictions. (**a**) Provides a historical time series and pandemic year forecasts, (**b**) provides a pandemic year data only.

Fracture episodes fell rapidly from the onset of the coronavirus (COVID-19) pandemic until April 2020, where observed episodes were 3,552 fewer than expected, before reversing the expected increase in episodes. Observed episodes remained lower than expected throughout the first period of national lockdown (Figure 1). Upon cessation of the first period of national lockdown, observed episodes rapidly returned to the expected range. This pattern was repeated during tiered lockdown restrictions through to lifting of the third national lockdown, with fracture episodes again falling though following the trend predicted in expected episodes. When the final period of national lockdown lifted, observed episodes rapidly returned to within the expected range. Observed episodes did not exceed the confidence intervals (95%) of expected episodes for the entirety of the study period.

**Figure 2:**
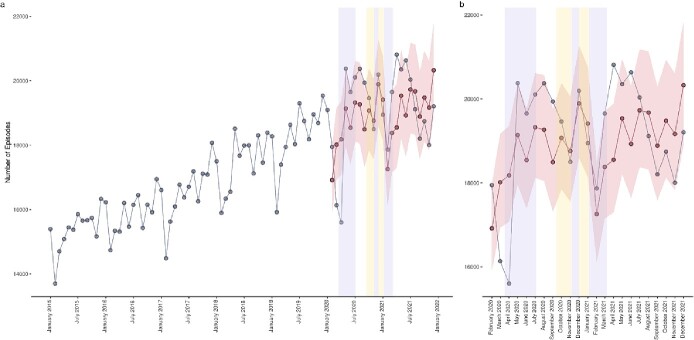
Predicted versus observed falls episodes. Blue data series indicate observed episodes, red data series indicate estimated episodes and their 95% confidence intervals, lilac shaded areas indicate periods of national lockdown restrictions and yellow shaded areas indicate periods of local tiered restrictions. (**a**) Provides a historical time series and pandemic year forecasts, (**b**) provides a pandemic year data only.

Fall episodes exhibited a similar trend to fracture episodes, diminishing from pandemic onset until April 2020 (Figure 2), where 2,842 fewer episodes were observed than expected. Observed episodes rapidly increased between April and May 2020, exceeding the expected number of episodes by 389. Fall episodes remained higher than estimated until onset of regional lockdown restrictions. Observed episodes tracked the trend of expected episodes for the duration of regional restrictions and the second and third instances of national lockdown, after which they exceeded expected episodes by 1,273. Observed episode frequency remained greater than expected until July 2021, with a transient dip below expected in May 2021. Like fracture episodes, fall episodes settled below the expected range from July 2021 to the end of the projection period.

**Figure 3:**
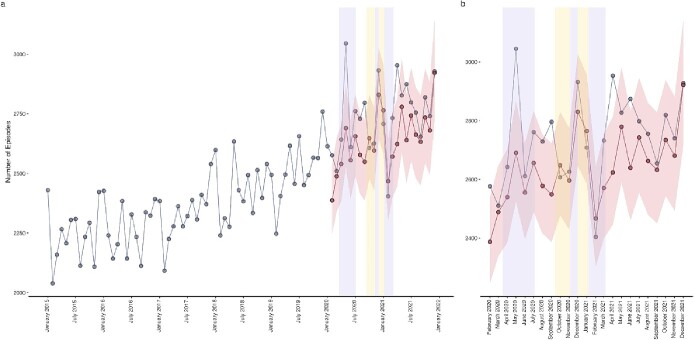
Predicted versus observed falls and fractures in those with frailty (‘frail falls and fractures’). Blue data series indicate observed episodes, red data series indicate estimated episodes and their 95% confidence intervals, lilac shaded areas indicate periods of national lockdown restrictions and yellow shaded areas indicate periods of local tiered restrictions. (**a**) Provides a historical time series and pandemic year forecasts, (**b**) provides a pandemic year data only.

Falls and fractures episodes for those designated as frail deviated from the trends observed in fracture and fall episodes in absence of a frailty marker (Figure 3). When frailty was considered, observed episodes peaked at 3,045 records in May 2020 during the first period of national lockdown, greatly exceeding the 2,634 estimated episodes. Observed episodes remained higher than estimated upon cessation of the first national lockdown, before peaking again during the second national lockdown at 2,932 episodes compared with the 2,772 expected. A third spike in episodes was evident on the lifting of the third period of national lockdown, suggesting a conferred increase in vulnerability to falls and fractures acquired during isolation. Contrary to fracture and fall episodes in absence of frailty markers, frail fall and fractures episodes settled above the expected trend.

High model accuracy was maintained across age stratifications except for fracture and frail fall and fracture patients <18 years old. Models were shown to be consistently accurate across regions (Supplementary materials, Table S2).

In age-stratified models, episodes for patients ≤18 years old tracked expected episodes (Supplementary materials, Figure S1). Within working-age and pension-age patients, differences between observed and estimated episodes were evident during the first period of national lockdown. Working-age episodes increased faster after the April 2020 minima than pension-age episodes. The magnitude of difference between observed and expected episodes during the second and third periods of national lockdown remained similar between these age groups.

Fall episodes also tracked estimated episodes in under 18s (Supplementary materials, Figure S2). The decrease visible in aggregated time series is therefore driven by working-age, pension-age and oldest (≥80) patients, with the largest decrease in the oldest. Fall episodes increased to May 2020 across all age groups, exceeding expected episodes in children and working-age adults. Fall episodes exceeded expected episodes in the months following both cessations of public health interventions (August 2020 and April 2021) in working-age, pension-age and oldest patients. These elevated episodes are suggestive of an increased vulnerability in these demographic groups after periods of potential increased isolation and decreased mobility.

**Figure 4:**
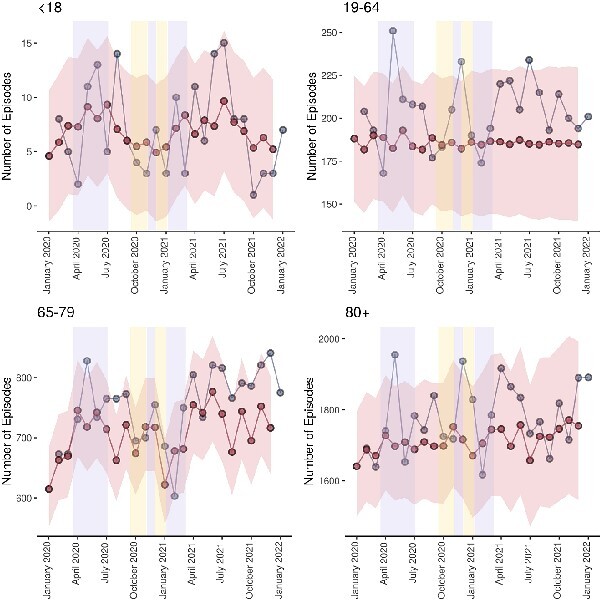
Predicted versus observed frail falls and fractures stratified by age category. Blue data series indicate observed episodes, red data series indicate estimated episodes and their 95% confidence intervals, lilac shaded areas indicate periods of national lockdown restrictions and yellow shaded areas indicate periods of local tiered restrictions.

Contrary to fall and fracture episodes, frail fall and fractures (Figure 4) remained higher than expected for working-age adults, pension-age adults and oldest adults for the majority of the time series, falling below this baseline for all groups, apart from the 65–79 age group, at the onset of the pandemic and during the third period of national lockdown. Observed episodes settled higher than the estimated baseline after curtailment of the third period of national lockdown in each of these age groups, with the largest difference evident in pension-age adults.

Stratification by region enabled impact of regional tiered restrictions to be more closely examined (Supplementary materials, Figure S3). An initial drop in fracture episodes at the onset of the pandemic was evident across all regions, with episode minima occurring in April 2020. In the East Midlands, London and the South East, greater disparities between observed and expected episodes were recorded at the transition between regional tiered restrictions and the onset of the third period of national lockdown. Observed episodes in the North East and West Midlands deviated from the national trend by exceeding the expected episodes levels in periods outside of public health restrictions.

The national trend in fall episodes appears to have been driven by the episodes in the North East, North West, West Midlands and East of England, where episodes exceeded expected levels in the first period of national lockdown and upon cessation of public health restrictions in April 2021 (Supplementary materials, Figure S4). They can be further attributed to the South East during the first lockdown period, and Yorkshire and the Humber post-April 2020. Greater absolute differences in episode numbers occurred in regions during or immediately following tiered public health restrictions, such as in November 2020 in the East Midlands and December 2020 in London and South East. Observed episode levels settled below the baseline of expected episodes in the North West, the South East and the South West.

There was no difference between sexes in observed versus expected admissions.

## Discussion

This study, which involved an analysis of 144,148,915 hospital records in England, found increased incidence of falls and fractures, beyond that expected based upon modelling, following periods of lockdown during the COVID-19 pandemic in older people. This was evident for those with frailty ascertained using the Hospital-wide Frailty Index but not for those without frailty. This suggests that, for those with frailty, the pandemic was associated with persistent increased risk of falls and fractures.

The rapid reduction in falls rates during lockdown was to be expected given the marked reduction in physical activity for most people and reduced exposure to outdoor hazards during these periods. Working-age fracture episodes increased faster after the April 2020 minima than pension-age episodes. This may be due to a phased return to work programme occurring before curtailment of public health interventions, which would expose working-age individuals to workplace risks that would be absent in pension-aged counterparts. Observed episodes for those aged ≥80 exhibited the greatest decrease until April 2020. Episodes for this group oscillated in their recovery to estimated levels in September 2020. This delay in recovery to predicted episode rates may suggest a hesitance to return to day-to-day activities after the lifting of lockdown restrictions, and hence reduced risk of falls and fractures. Observed episodes for falls and fractures settled higher than the estimated baseline after curtailment of the third period of national lockdown in working-age-and-above age groups, with the largest difference evident in pension-age adults. This perhaps correlates with the sustained elevated risk of fall or fracture within frail individuals within this demographic.

Additional regional lockdown restrictions, over and above those imposed in other parts of England, took place in the East Midlands, London and the South East. In these regions, greater disparities were seen between observed and estimated episodes at the transition between regional tiered restrictions and the onset of the third period of national lockdown. Observed fall episode levels settled below the baseline of estimated episodes in London, the South East and the South West, suggesting a lasting impact of public health interventions on episode levels in these areas. These regional differences are, however, difficult to interpret because the numbers are smaller and because of variance in coding practices across hospitals and in the social care response during the pandemic between regions. In addition, the granularity of the data does not fully capture the areas affected by additional lockdowns—it was not, for example, the whole East Midlands that was affected by tiered lockdown, but only the City of Leicester. They serve mainly to highlight that the pandemic was experienced differently in different regions, and to show that this, to some extent, correlated with policy decisions regarding lockdown and social isolation.

Frailty has been described as resulting from cumulative declines across multiple physiologic systems, causing vulnerability to adverse outcomes [22]. Whether viewed as multisystem homeostatic compromise or simply as the straightforward accumulation of deficits, a key concept is that frailty is a cascade, which progresses from mild through moderate to severe frailty [23]. It is unsurprising that the patient group most at risk of adverse outcomes in this study were those with pre-existing frailty. A number of studies conducted during the pandemic have shown that frailty is an independent predictor of adverse outcomes following COVID-19 infection, including death, disability and admission to higher levels of care [24, 25]. This study adds to this literature by showing that, for those with frailty, there was an increased risk of other indirect adverse events—namely falls and fractures. This increased vulnerability likely relates in part to acute effects and sequelae of COVID-19 infection in some of these individuals—older adults have been shown to have worse mobility and functional outcomes following a COVID-19 diagnosis, even where they did not require hospitalisation [26]. Social isolation, immobility and physical deconditioning are also likely contributors. Data from the Sport England Active Lives study showed reduced duration of physical activity, particularly strength and balance training, amongst older people during March and May 2020 and modelling suggested that this would result in an increase in falls of 3.9% amongst those aged ≥65 [27]—so it is at least plausible that deconditioning played a role. Older people who are less active already have a higher risk of falls [28] and this group are most likely to have been using community rehabilitation services and/or care services to maintain mobility at the start of the pandemic that subsequently stopped during lockdown. Each of these issues is likely to have interacted with and been compounded by, reduced access to paid and unpaid carers during periods of lockdown [29]. Although there was some success with moving rehabilitation services and exercise programmes online during the pandemic [30], older people with frailty were most likely to find accessing such approaches difficult [31]. Specific strategies to maintain activity and social participation in the context of the pandemic might have mitigated against some these adverse events and should be a specific consideration in future pandemic preparedness, taking account of the risks of digital exclusion for those with frailty.

The strengths of this study relate to the large and representative national datasets covering the whole of England, and the length of historical data available pre-pandemic enabling robust modelling of predicted adverse outcome rates. Limitations relate to the extent to which falls and fractures can be relied upon as proxy indicators of frailty and disability, and the reliability with which the HFRS determines frailty. The HFRS is a measure of frailty risk, which correlates at best modestly with other frailty measures (19)—it was, however, the only widely used frailty score readily calculable using our dataset and is well validated, and we applied it carefully according to protocols laid out in validation papers. Using concurrent data from the fracture admission, but not from earlier hospital admissions, to calculate HFRS may have resulted in under-detection of frailty, so that some people with frailty were excluded from the frail falls and fracture cohort. However, because we applied this consistently across the training, validation and pandemic periods, it is unlikely that this substantially influenced the inferences drawn about falls and fracture incidences for those with and without frailty over time. Hospital diagnostic codes have some limitations. Patients who switch consultants or provider organisations during episodes may have the same admission recorded as two episodes—but there is no reason to suspect that transfers between consultants or providers differed substantively in frequency from usual during the pandemic. Patients who presented with a fall or fracture during the pandemic who also tested positive for SARS-CoV-2, either at the point of admission or subsequently, may have had COVID-19 recorded as their primary diagnosis, given the focus on accurate coding of COVID-19. However, we captured data on secondary diagnoses as part of our analysis, which would have mitigated against this to some extent. Differences in coding practices between hospitals, and of local practice within social care, render regional comparisons heavily caveated but are unlikely to have influenced national-level findings. We are not aware of any substantial or systematic changes to coding practice around falls and fractures before, during or after the pandemic that could have biassed findings. There is some overlap in the codes used to diagnose falls and fractures in this study, and fracture codes included in the HFRS. However, because fractures are the outcome of interest and HFRS was used to identify a sub-cohort of fallers with fractures, this will not have introduced bias. An important additional limitation is the concept of mortality as a competing risk. People who died of COVID could not go on to experience subsequent falls or fractures. The group of people with frailty for whom we found sustained increased fall and fracture risk, though, are also amongst the population groups that experienced the highest excess mortality during the pandemic, particularly during the first wave in Spring 2020—so competing risks are unlikely to explain why there was a sustained increased fall and fracture risk. Finally, we did not include minor falls and fractures seen in emergency departments but not admitted to hospital.

In conclusion, during the COVID-19 pandemic (2020–22) there were deviations from expected fracture admission rates for all population groups. Older people with frailty experienced an elevated and persistently increased risk of falls and fractures for the duration of the COVID-19 pandemic by comparison with the rates based upon modelling using historical data. Further research is required to unpick the relative contributions of COVID-19 infection versus social isolation, immobility and deconditioning. These findings add further weight to the assertion that this population group deserves specific consideration and provision as part of future pandemic preparedness planning.

## Supplementary Material

aa-23-1653-File002_afae071

## Patient and Public Involvement:

This protocol was extensively discussed at a meeting of the East Midlands Research in Ageing Network, which includes patients, carers and public representatives, many of whom are older or care for older people with frailty. The group was supportive of the aims and was particularly keen to understand whether COVID-19-associated lockdown could have been detrimental to older people with COVID-19 during the pandemic. We are presenting findings at forthcoming meetings of the group and will use their input to shape lay-facing dissemination materials based upon the research.

## STROBE Checklist:

This paper has been reported in line with the requirements of Strengthening the Reporting of Observational Studies in Epidemiology (STROBE) checklist. This is published in the supplementary materials.

## Dissemination Statement:

The work outlined in this paper has been made publicly available, in abbreviated form, on the website of the Office of National Statistics (ONS), in keeping with the ONS transparency and governance framework. This version of the findings can be accessed here:

## Data Sharing Statement:

All data for this project have been supplied to ONS under a data sharing agreement (DSA) with the respective data owner, which outlines the legal basis for data sharing and the permitted purposes for data use. Each individual dataset has an ONS Information Asset Owner (IAO) who is accountable for data management and use throughout the end-to-end data life cycle. ONS has an established data management, retention and disposal processes in place to ensure all data are only kept for as long as necessary, in line with the relevant DSA conditions and legal requirements. Access to data is granted only for the purposes of an approved, ethically assessed project, subject to IAO agreement and is via a restricted ONS data processing and statistical analysis environment with physical, technical and administrative controls applied in line with UK government security standards.
