# Supplementary material for: Impact of the COVID-19 pandemic on hospital episodes for falls and fractures associated with new-onset disability and frailty in England: a national cohort study
Source: Age Ageing. 2024 Apr 6;53(4):afae071. doi: 10.1093/ageing/afae071 (PMC10998734; doi:10.1093/ageing/afae071)
Supplement: aa-23-1653-File002_afae071 [file aa-23-1653-file002_afae071.docx]

# Supplementary materials

Supplementary Table 1 - ICD-10 diagnostic codes used to classify falls and fractures

| Fractures were defined using codes from Chapter 19 of the ICD-10 focused on commonly experienced fractures by older people | |
| --- | --- |
| ICD-10 code | Descriptor |
| S22.3 | Fracture of rib |
| S22.4 | Multiple fractures of ribs |
| S52.5 | Fracture of lower end of radius |
| S72.0 | Fracture of femur |
| Falls were defined using the following ICD-10 codes | |
| W01.0 | Fall on same level from slipping, tripping and stumbling |
| W03.0 | Other fall on same level due to collision with, or pushing by, another person |
| W05.0 | Fall involving wheelchair |
| W06.0 | Fall involving bed |
| W08.0 | Fall involving other furniture |
| W18.0 | Other fall on same level |
| W19.0 | Unspecified fall |

Supplementary Table 2 - Model Accuracy by event type across whole nation, and stratified by age category and region (AICc=Corrected Akaike’s Information Criterion; MAPE=Mean Absolute Percentage Error)

| **Episode Cause** | **Stratification** | **Model Parameters** | **AICc** | **MAPE** |
| --- | --- | --- | --- | --- |
| Fractures | National | ARIMA(1, 0, 1), (2, 1, 0) | 1478.55 | 2.56% |
| Falls | National | ARIMA(1, 0, 1), (1, 1, 0) | 1070.02 | 1.41% |
| Frail Fall and Fracture | National | ARIMA(0, 1, 1), (1, 1, 1) | 860.41 | 2.29% |
| Fracture | Children (< 18) | (0, 0, 0), (0, 1, 2) | 1165.84 | 10.90% |
|  | Working Age (19-64) | (0, 1, 1), (0, 1, 1) | 1242.97 | 3.28% |
|  | Pension Age (65-79) | (0, 1, 1), (2, 0, 0) | 1406.87 | 3.34% |
|  | Elderly (80+) | (0, 1, 1), (2, 0, 0) | 1492.47 | 2.83% |
| Fall | Children (< 18) | (0, 0, 0), (1, 1, 0) | 785.5 | 3.94% |
|  | Working Age (19-64) | (1, 0, 0), (1, 1, 0) | 873.67 | 2.91% |
|  | Pension Age (65-79) | (1, 0, 1), (0, 1, 1) | 912.49 | 1.91% |
|  | Elderly (80+) | (0, 0, 0), (0, 1, 1) | 1115.69 | 3.53% |
| Frail Fall and Fracture | Children (< 18) | (1, 0, 0), (2, 0, 0) | 442.94 | 53.82% |
|  | Working Age (19-64) | (1, 1, 1), (1, 0, 0) | 738.53 | 9.12% |
|  | Pension Age (65-79) | (0, 0, 0), (0, 1, 1) | 730.37 | 3.75% |
|  | Elderly (80+) | (0, 1, 3), (1, 1, 0) | 824.35 | 3.03% |
| Fracture | North East | (0, 1, 1), (2, 0, 0) | 1054.4 | 5.53% |
|  | North West | (0, 1, 1), (2, 0, 0) | 1158.44 | 4.28% |
|  | Yorkshire and The Humber | (0, 1, 1), (2, 0, 0) | 1144.66 | 5.24% |
|  | East Midlands | (0, 1, 1), (0, 0, 1) | 1131.29 | 6.06% |
|  | West Midlands | (0, 1, 4), (0, 0, 2) | 1128.27 | 4.57% |
|  | East of England | (0, 1, 1), (0, 0, 2) | 1130.8 | 4.69% |
|  | London | (0, 1, 1), (1, 0, 1) | 1101.49 | 3.82% |
|  | South East | (1, 1, 2), (0, 0, 2) | 1204.8 | 4.31% |
|  | South West | (0, 1, 1), (0, 1, 1) | 1000.69 | 3.58% |
| Fall | North East | (0, 1, 1), (1, 0, 2) | 888.95 | 3.95% |
|  | North West | (0, 0, 1), (0, 1, 1) | 857.77 | 2.30% |
|  | Yorkshire and The Humber | (1, 0, 1), (2, 1, 0) | 836.75 | 2.68% |
|  | East Midlands | (0, 1, 2), (0, 0, 2) | 984 | 4.23% |
|  | West Midlands | (1, 0, 1), (1, 0, 0) | 981.24 | 3.53% |
|  | East of England | (0, 0, 2), (0, 1, 1) | 848.41 | 2.34% |
|  | London | (0, 1, 1), (1, 0, 1) | 974.71 | 2.99% |
|  | South East | (0, 1, 2), (2, 0, 0) | 1022.87 | 2.63% |
|  | South West | (0, 1, 1), (2, 0, 0) | 975.98 | 3.09% |


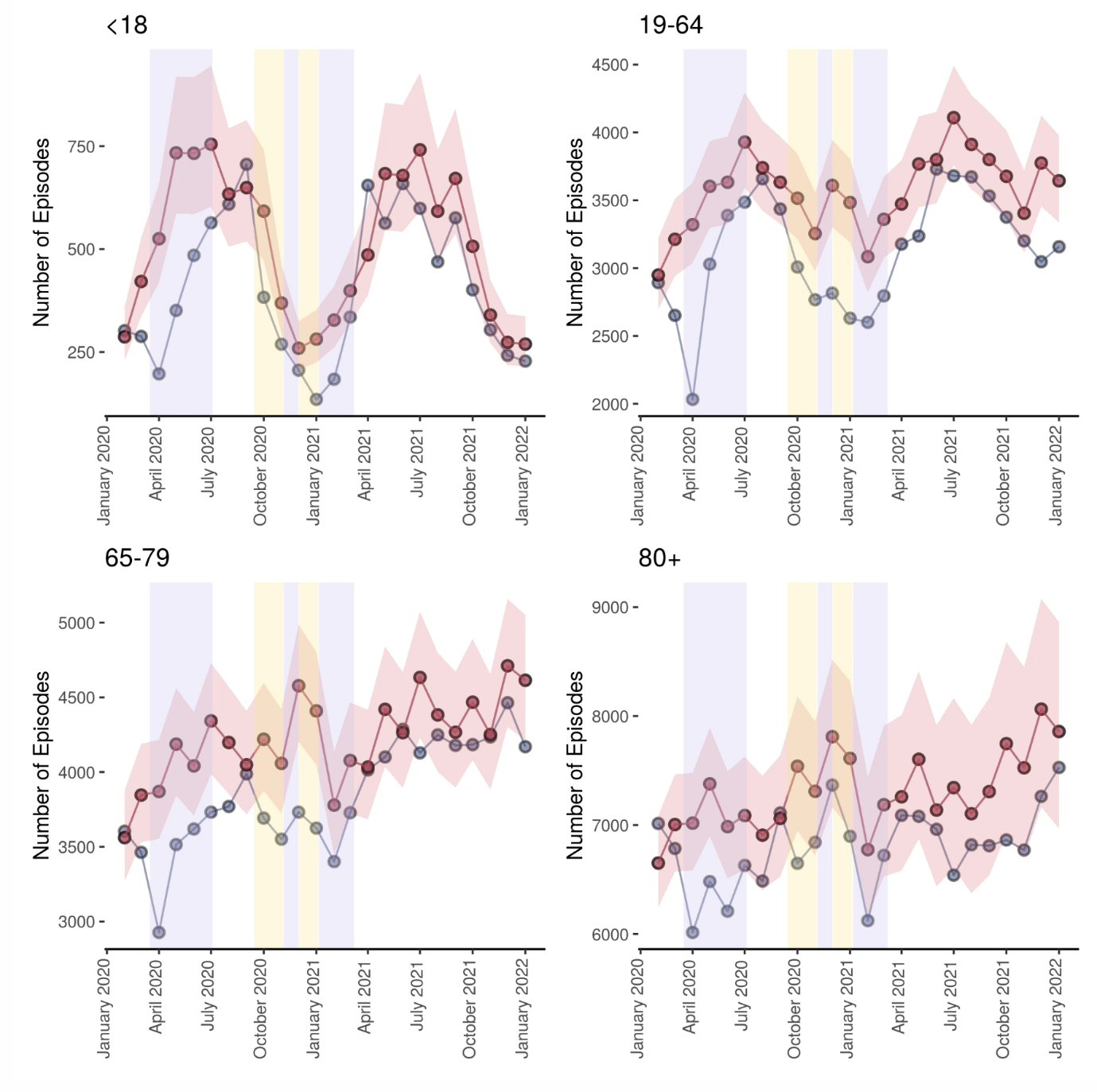


Supplementary Figure 1 - Fracture episodes predicted vs observed stratified by age category

*Blue data series indicate observed episodes, red data series indicate estimated episodes and their 95% confidence intervals, lilac shaded areas indicate periods of national lockdown restrictions and yellow shaded areas indicate periods of local tiered restrictions. Scales differ on axis due to the differences in incidence by age.*


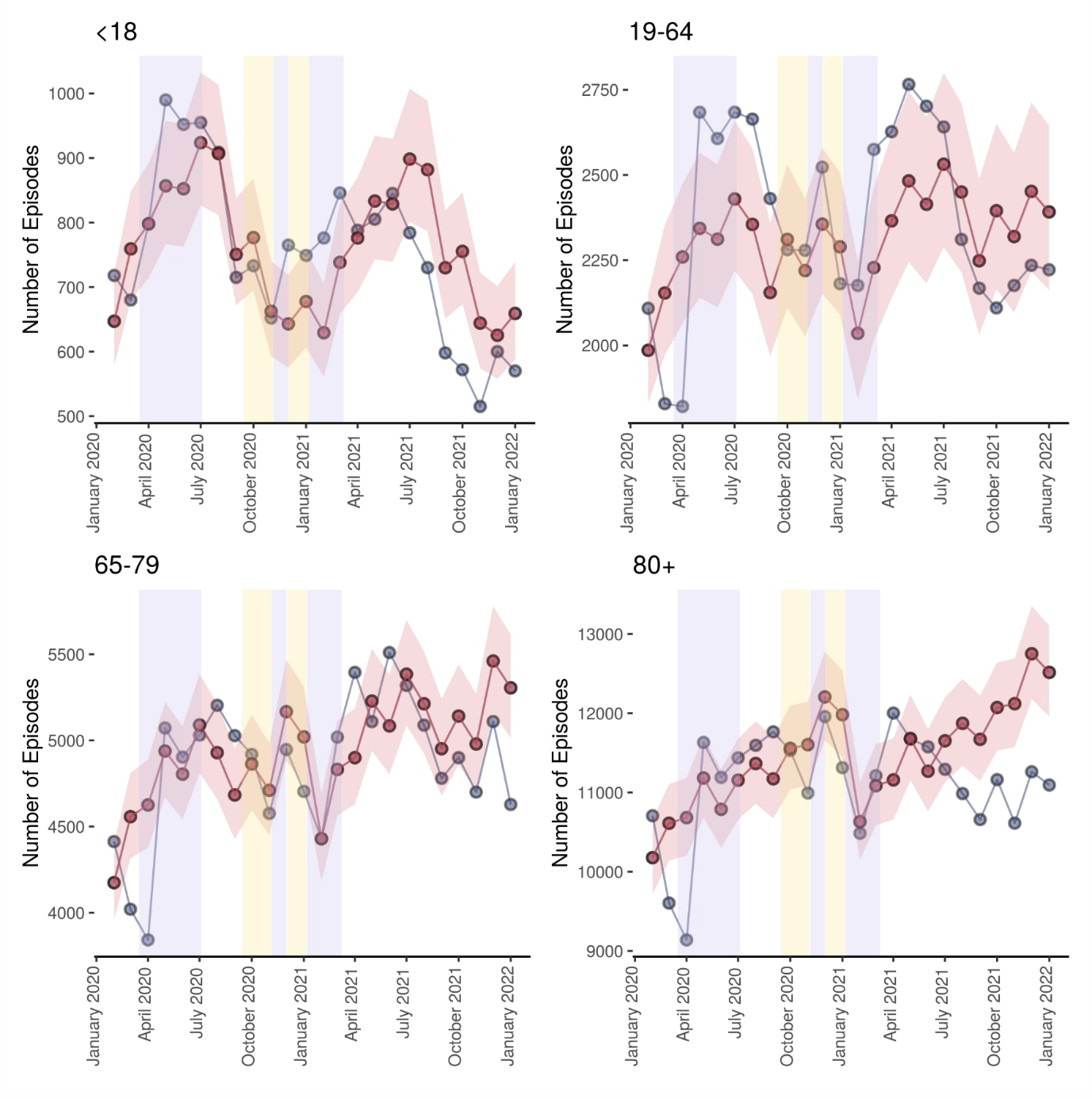


Supplementary Figure 2 - Falls episodes predicted vs expected by age category

*Blue data series indicate observed episodes, red data series indicate estimated episodes and their 95% confidence intervals, lilac shaded areas indicate periods of national lockdown restrictions and yellow shaded areas indicate periods of local tiered restrictions.*


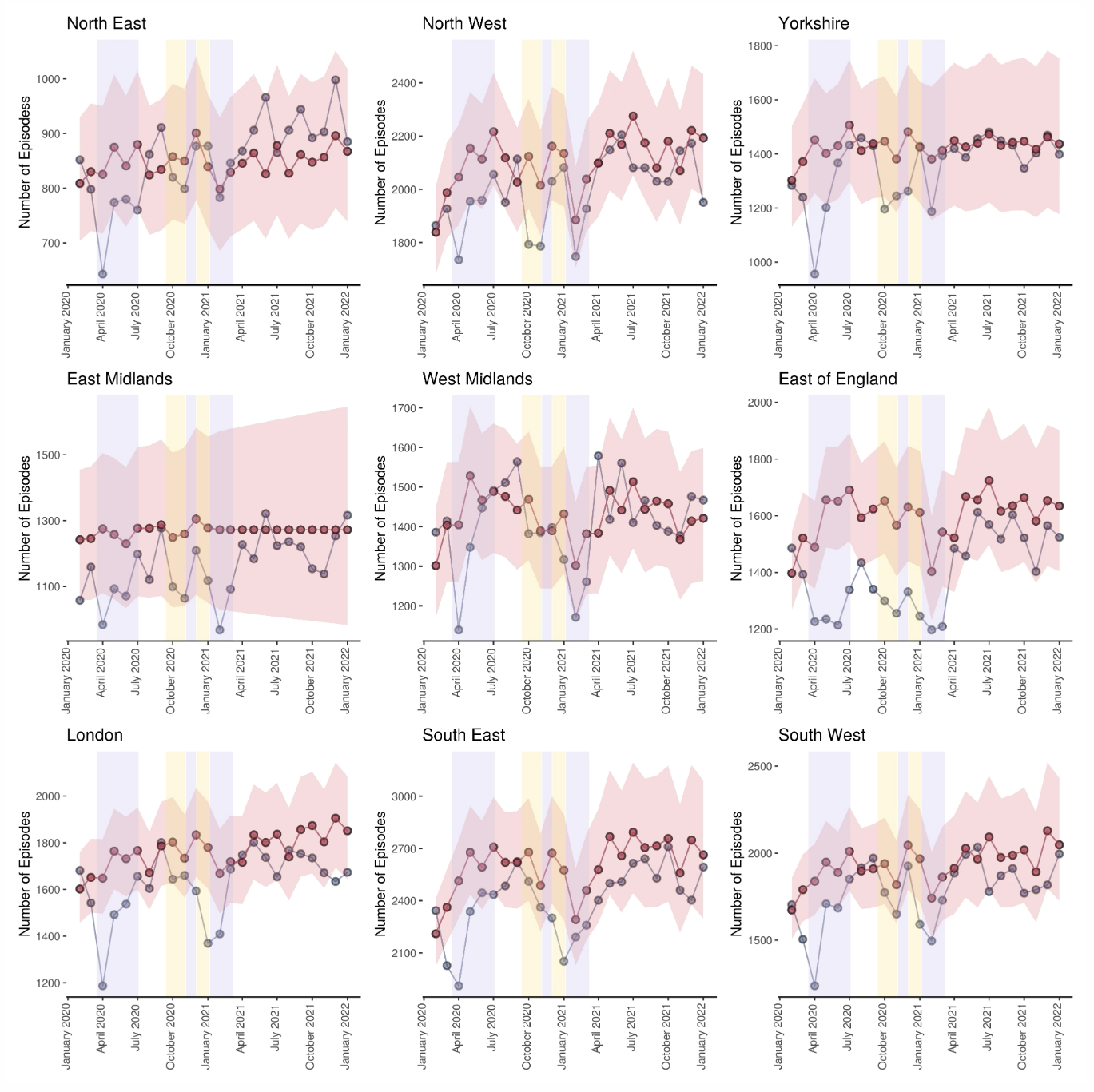


Supplementary Figure 3 - Predicted vs observed fractures stratified by region

*Blue data series indicate observed episodes, red data series indicate estimated episodes and their 95% confidence intervals, lilac shaded areas indicate periods of national lockdown restrictions and yellow shaded areas indicate periods of local tiered restrictions.*


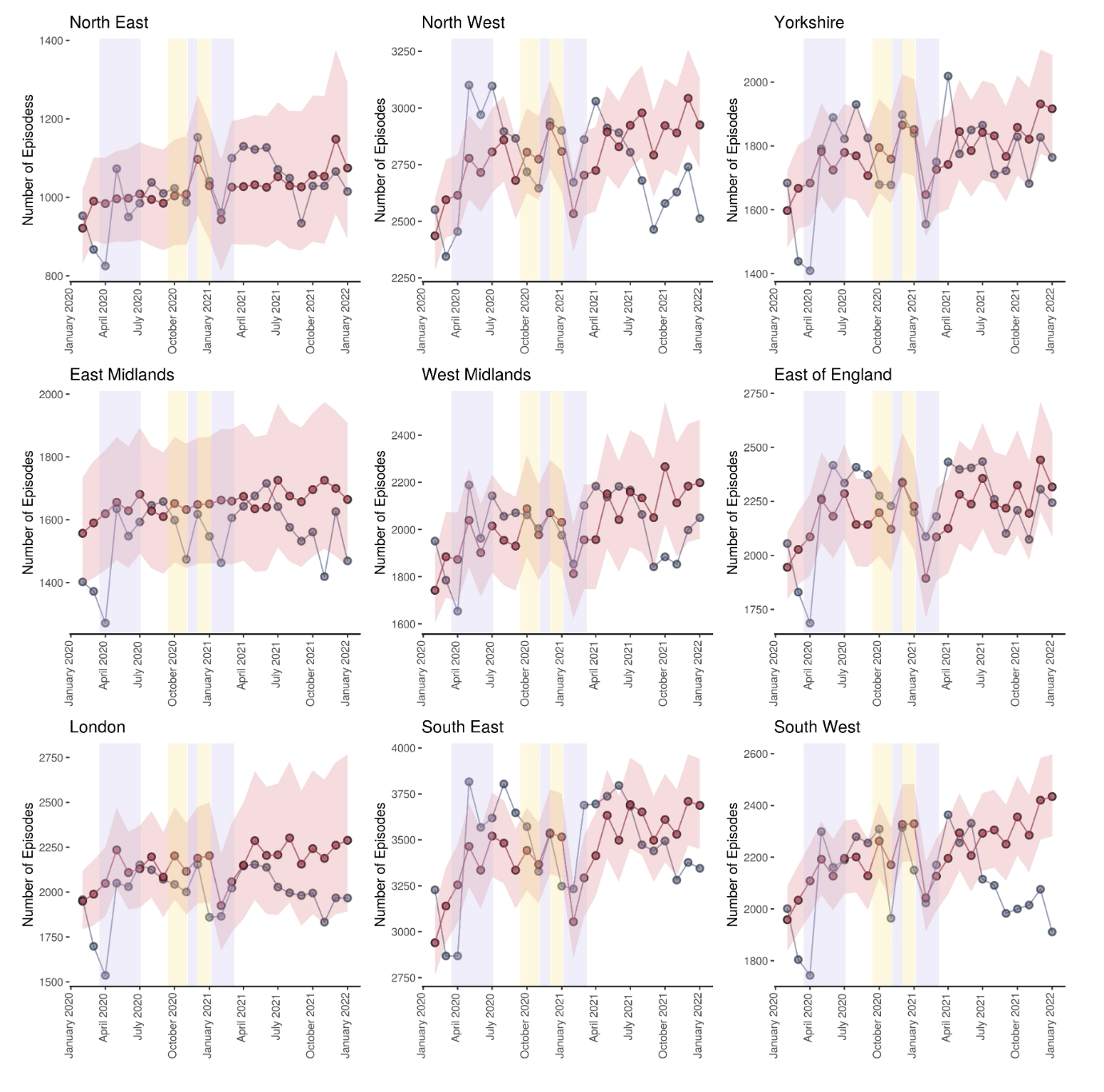


Supplementary Figure 4 - Predicted vs expected falls episodes stratified by region

*Blue data series indicate observed episodes, red data series indicate estimated episodes and their 95% confidence intervals, lilac shaded areas indicate periods of national lockdown restrictions and yellow shaded areas indicate periods of local tiered restrictions.*
